# COVID-19 Nursing Home Deaths in Cook County: Predictors and Modifiable Risks

**DOI:** 10.1093/geroni/igab046.2011

**Published:** 2021-12-17

**Authors:** Andrew DeMott, Susan Hughes, Michael Gelder, Sage Kim

**Affiliations:** 1 UIC, Chicago, Illinois, United States; 2 University of Illinois at Chicago, Chicago, Illinois, United States

## Abstract

Investigators at the University of Illinois Chicago, the Illinois Department on Aging (IDOA), the Illinois Department of Public Health, and the Health and Medicine Policy Research Group are collaborating to examine comparative rates of Covid-19-related deaths among older adults who reside in nursing homes vs. the community in Illinois. As a first step, we have examined data from the Cook County Medical Examiner’s office to compare nursing home resident fatalities to those who died in the community. Deaths with Covid-19 listed as primary or secondary cause of death that occurred between January 1, 2020 to September 30, 2020 among older adults ages 60 and over were identified from the Cook County Medical Examiner’s Office case archive file. Location at death and race/ethnicity were obtained from the same source. Location at death was matched with data in the Center for Medicare and Medicaid Services (CMS) Covid-19 Nursing Home Data to identify persons who died in skilled nursing facilities (SNFs) as well as facility and staff characteristics. We found that the 3,937 deaths among persons over the age of 60 comprised 75% of total deaths in Cook County. Of the total older adult deaths, 2,090 (53%) died in the community and 1,837 (47%) died in SNFs. Regression analyses that controlled for CMS quality ratings found that larger, for-profit nursing homes, with high levels of staff infected with Covid-19 were associated with higher mortality. The policy implications of these findings will be discussed.

